# Supplementary material for: Interpretable Structural Evaluation of Metal-Oxide Nanostructures in Scanning Transmission Electron Microscopy (STEM) Images via Persistent Homology
Source: Nanomaterials (Basel). 2024 Aug 29;14(17):1413. doi: 10.3390/nano14171413 (PMC11397156; doi:10.3390/nano14171413)
Supplement: Supplementary file 1 [file nanomaterials-14-01413-s001.zip › nanomaterials-3139941-supplementary.pdf]

nanomaterials

Supporting Information

Interpretable Structural Evaluation of Metal-Oxide Nanostructures in Scanning Transmission Electron Microscopy (STEM) Images via Persistent Homology

Ryuto Eguchi [1,2,*], Yu Wen [1,2], Hideki Abe [1,3] and Ayako Hashimoto [1,2,*]

1 National Institute for Materials Science, 1-2-1 Sengen, Tsukuba 305-0047, Ibaraki, Japan; yu.wen.lw@alumni.tsukuba.ac.jp (Y.W.); ABE.Hideki@nims.go.jp (H.A.)
2 Graduate School of Pure and Applied Sciences, University of Tsukuba, 1-2-1 Sengen, Tsukuba 305-0047, Ibaraki, Japan
3 Graduate School of Science and Engineering, Saitama University, Shimo-Okubo 255, Saitama 338-8570, Japan
* Correspondence: EGUCHI.Ryuto@nims.go.jp (R.E.); HASHIMOTO.Ayako@nims.go.jp (A.H.)

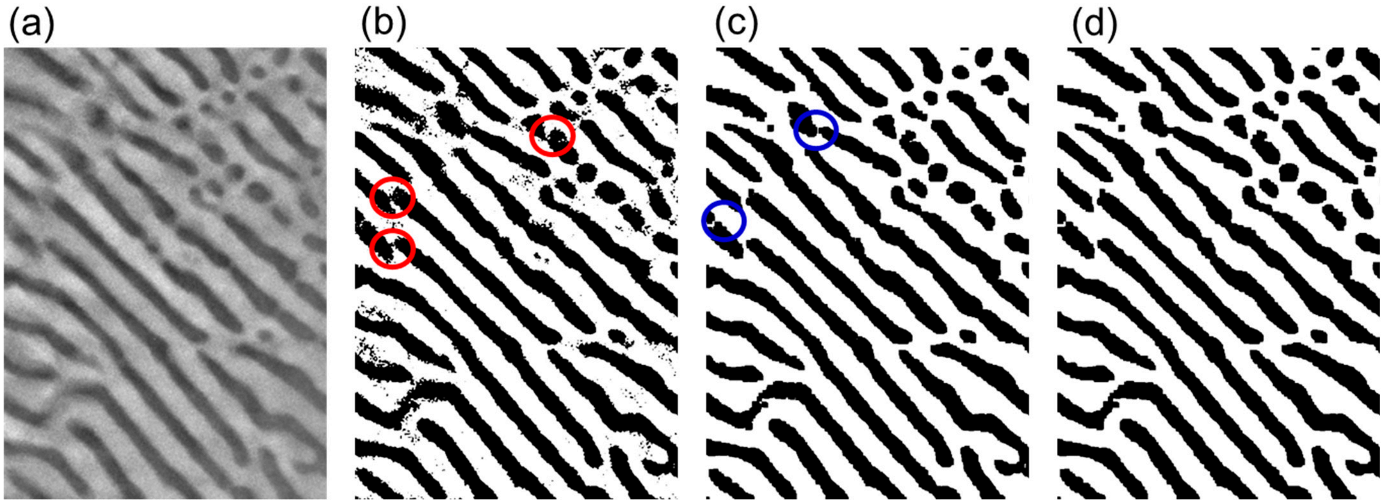

Figure S1. Binarization and post-processing of the background removal image for the STEM images before the PH analysis. (a) The background-removed image by fast Fourier transform; (b) corresponding binarized image using an adaptive Gaussian thresholding method; (c) closing, where unnecessary connections of black parts marked by red circles and black spots in (b) were removed; (d) opening, where gaps in black parts marked by blue circles in (c) were filled and white noise was removed. The processes had two parameters; block size in an adaptive Gaussian thresholding method, and kernel size in an opening-closing process. A number of the binarized images were produced by changing them, to calculate the ratios between the white to black areas. From the ratio of Pt and Ce in the Pt_5Ce alloy precursor, the volume ratios of Pt and CeO_2 phases, that is, the ideal ratios of the white and black domains were obtained. The parameters of the binarized images whose ratio was similar with the ideal area ratio (block size is 99 and kernel size is 3) were employed for all the 84 STEM images. Reproduced from reference [15] with the permission of AIP Publishing.

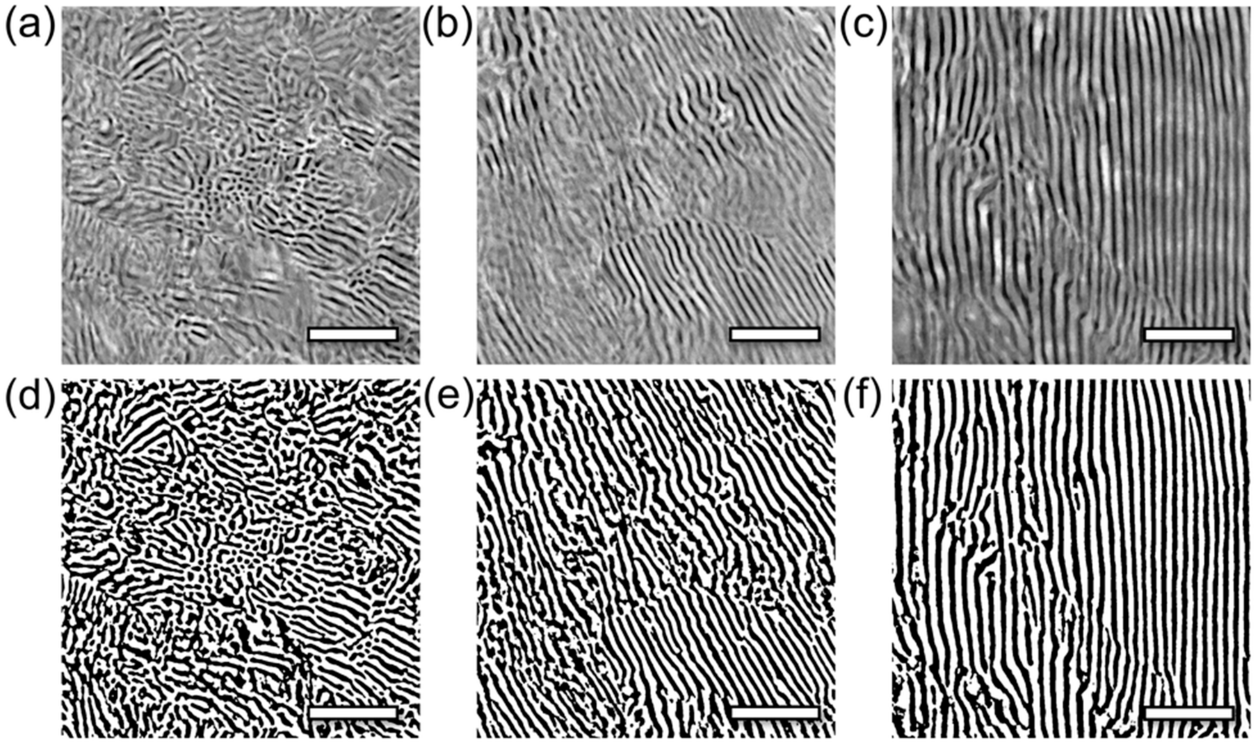

Figure S2. Original HAADF-STEM images of Pt-CeO_2 nanostructures synthesized at (a) 500 ℃, (b) 600℃, and (c) 700℃ with a CO:O_2 gas ratio of 2:1. (d), (e), and (f) Binarized images corresponding to (a), (b), and (c), respectively, which are identical to Figure 2(a)-(c). Scale bars represent 100 nm.

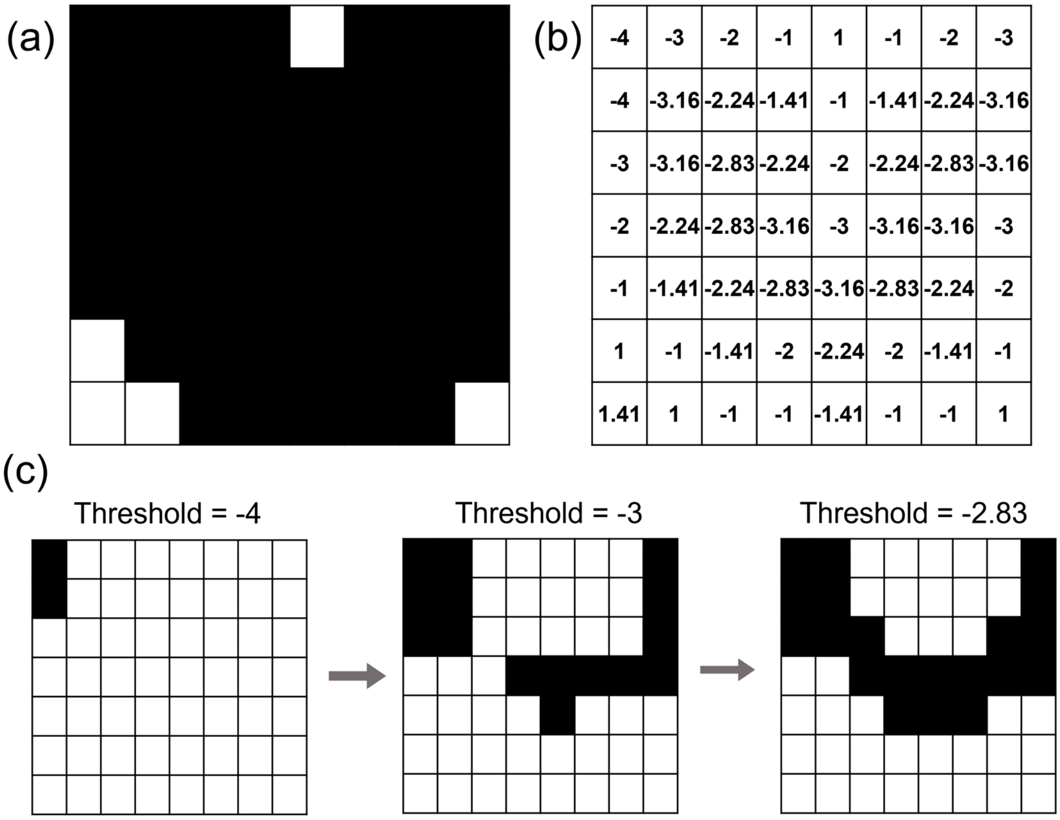

Figure S3. Assignment of distance and filtration for zero-dimensional holes in binary image contrast. (a) Black domain in a binarized image and (b) corresponding signed Euclidean distance (SED) for each pixel. The SED signs were set negative for the black domains and positive for the white. The absolute values are the distance from the boundaries between the black and white domains. (c) Filtration process for (a) by increasing the threshold for binarization persisting from minimum to maximum SED values. For example, as the black domain appears at a threshold of −4 and disappears at −2.83, the *b*-*d* point (*b*, *d*) is (−4, −2.83) and lifetime (*d*–*b*) is 1.17. The *b*-*d* points are plotted two-dimensionally for all domains in the obtained binarized images as the zeroth persistent diagram (PD).

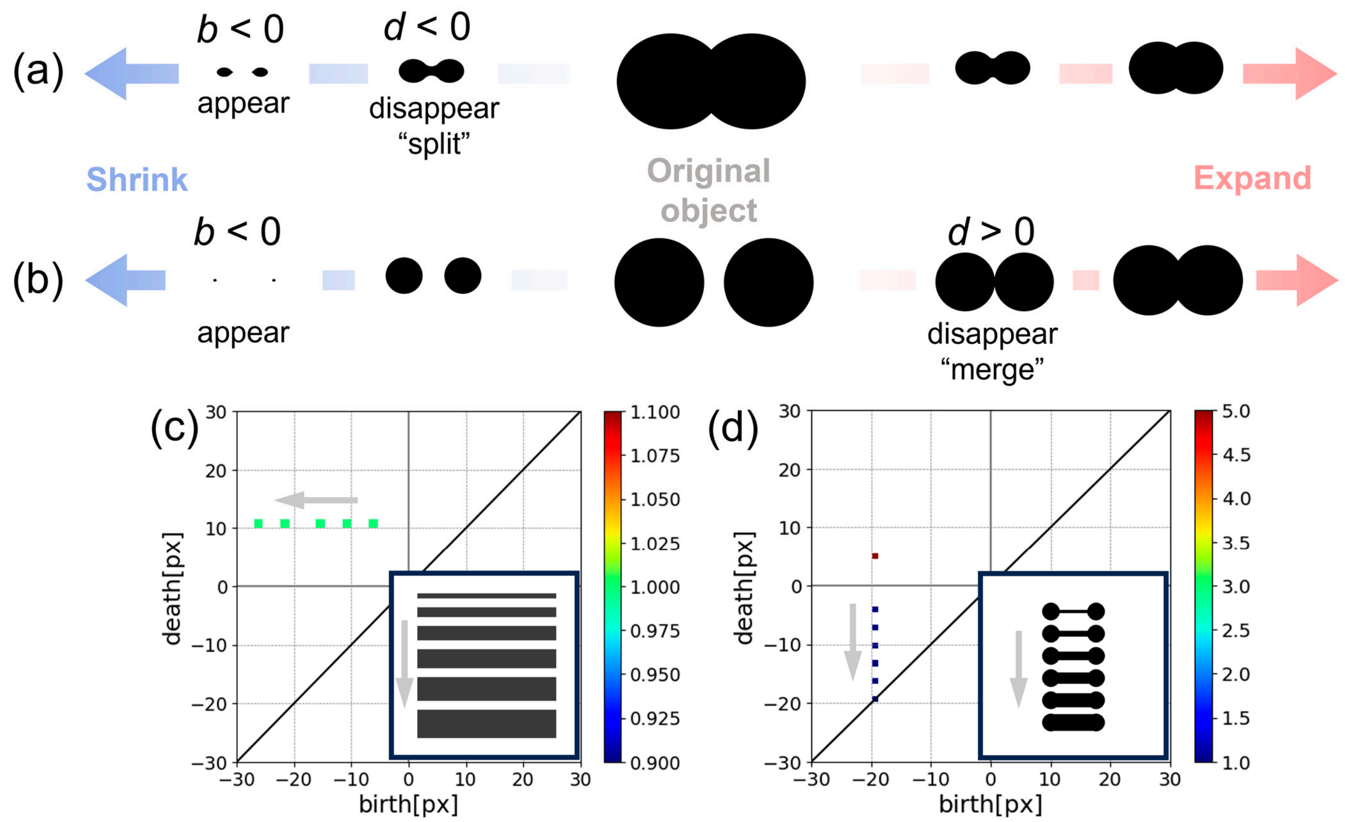

Figure S4. Filtration processes of (a) connected and (b) isolated domains for the zeroth PDs. The red and blue arrows indicate the expansion and shrinkage of the original objects caused by the filtration, respectively. Each death (*d*) in (a) and (b) is represented as splitting during shrinkage and merging during expansion, respectively. (c) and (d) show zeroth PDs of two kinds of representative object shapes (inset). (c) PD of six stripes, each widening by 10 px with uniform interval. Basing on the filtration process, the domain for the stripe appears when a stripe-shaped domain shrinks by half of the stripe width. Thus, the minimum SED for birth (*b*) corresponds to half of the stripe width. The domain for the stripe merges five times, which is equal to the number of intervals. Thus, the number of *b*-*d* pairs corresponds to the number of isolated domains minus one. (d) PD of six dumbbell-like structures composed of two circles with a diameter 38 px connected by bridges that widened by 6 px between neighboring structures. There is a 40 px spacing between each stripe. As the bridge width approaches the circle diameter, that is, the structure changes from dumbbell-shaped to striped, the *b*-*d* pairs approach $b = d$ (i.e., the diagonal line in the PD). In the Pt-CeO_2 nanostructures, although the width of the CeO_2 phase is inhomogeneous, *b*-*d* pairs of the striped structures were mainly located near the $b = d$ line. Therefore, the *b*-*d* pairs located less than 1.0 nm from the $b = d$ line (lifetime below 1.0 nm) were used to measure the width of CeO_2 stripes.

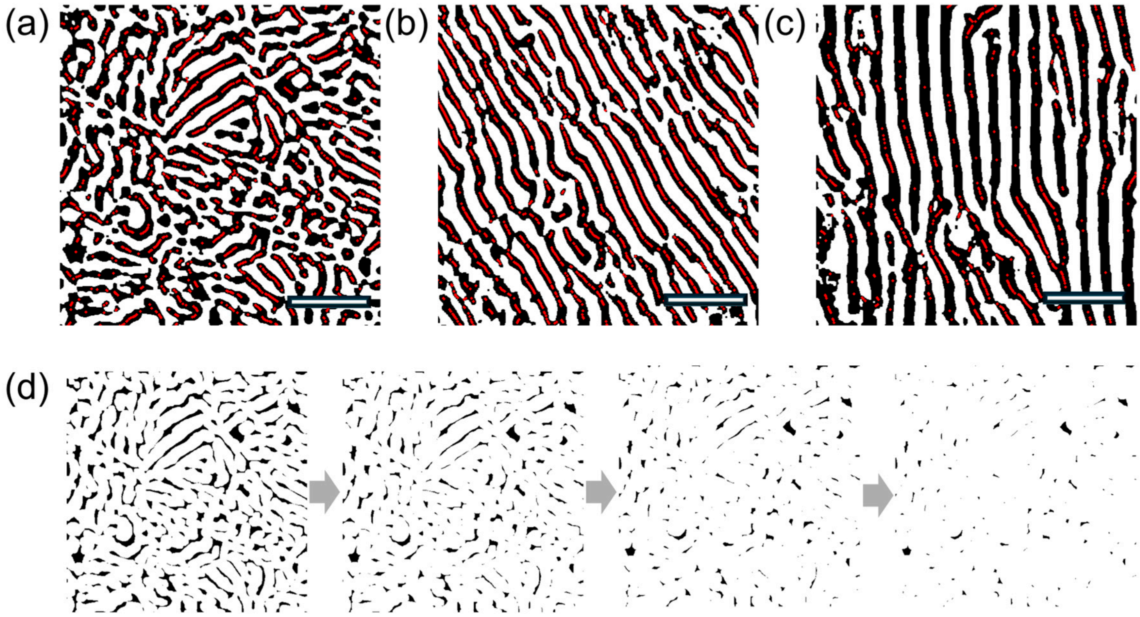

Figure S5. (a)–(c) Binarized HAADF-STEM images of Pt-CeO_2 nanostructures (the same as in Figure 1(a)–(c)) with birth points of the zeroth PDs (Figure 1(d)–(f)) with lifetimes shorter than 0.2 nm (indicated by red points). Scale bars represent 50 nm. (d) Filtration process for the nanostructure in (a). The splitting and appearance of small domains within the CeO_2 stripes can be seen during filtration, which was caused by the inhomogeneity of the stripe width. The inhomogeneous width of the CeO_2 stripes led to a distribution of numerous *b*-*d* pairs with a certain lifetime. Therefore, the number of *b*-*d* pairs with a short lifetime (< 0.2 nm) was used to measure the length of the CeO_2 stripes.

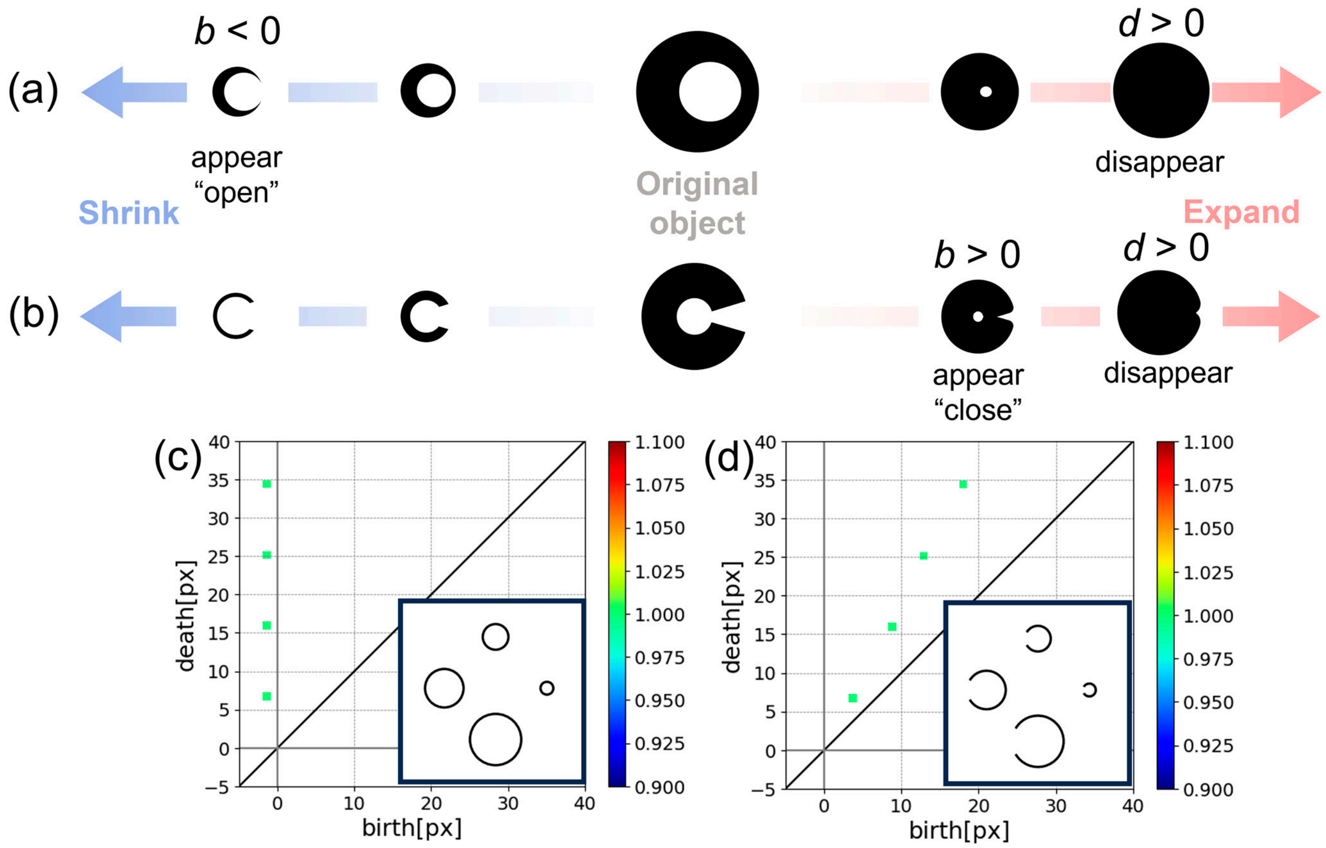

Figure S6. Filtration process of a (a) ring and (b) arc for the first PDs. The red and blue arrows indicate the expansion and shrinkage of the original objects caused by the filtration, respectively. Each birth in (a) and (b) is represented as "open" during shrinkage and "closed" during expansion, respectively. First PDs of four representative (c) ring and (d) arc shapes (inset). (c) PD of four rings with increasing diameter by 20 px with an identical interval from each ring center. On the basis of the filtration process, the circle disappeared when it expanded by half of the diameter; i.e., the ring radius. (d) PD of four arcs corresponding to arcs each forming a 300° segment with increasing diameter by 20 px with an identical interval from each arc center. The number of *b*-*d* pairs corresponded to that of rings and arcs because it corresponded to the frequency of their disappearance.

Table S1. Mean cosine similarities and their deviations of vectorized PDs for Pt-CeO_2 nanostructures synthesized under different annealing conditions. The mean cosine similarity was calculated from 21 pairs of vectorized PDs among the seven images of the nanocomposite fabricated under the same set of conditions. The mean deviation was calculated from the error for the 21 cosine similarities determined for the same set of conditions. We used the maximum deviation of 0.007148 determined for the conditions of an annealing temperature of 600 °C and syngas ratio of 2:1 as the threshold for cluster formation.

Temperature, gas ratios	Mean cosine similarity	Mean deviation
500℃, 0:1	0.999229	0.000622
500℃, 1:1	0.999619	0.000348
500℃, 2:1	0.996067	0.004096
500℃, 3:1	0.996019	0.002913
600℃, 0:1	0.998746	0.000928
600℃, 1:1	0.997993	0.002627
600℃, 2:1	0.993117	0.007148
600℃, 3:1	0.991114	0.004854
700℃, 0:1	0.998957	0.001058
700℃, 1:1	0.994674	0.005567
700℃, 2:1	0.995880	0.002111
700℃, 3:1	0.997784	0.002137

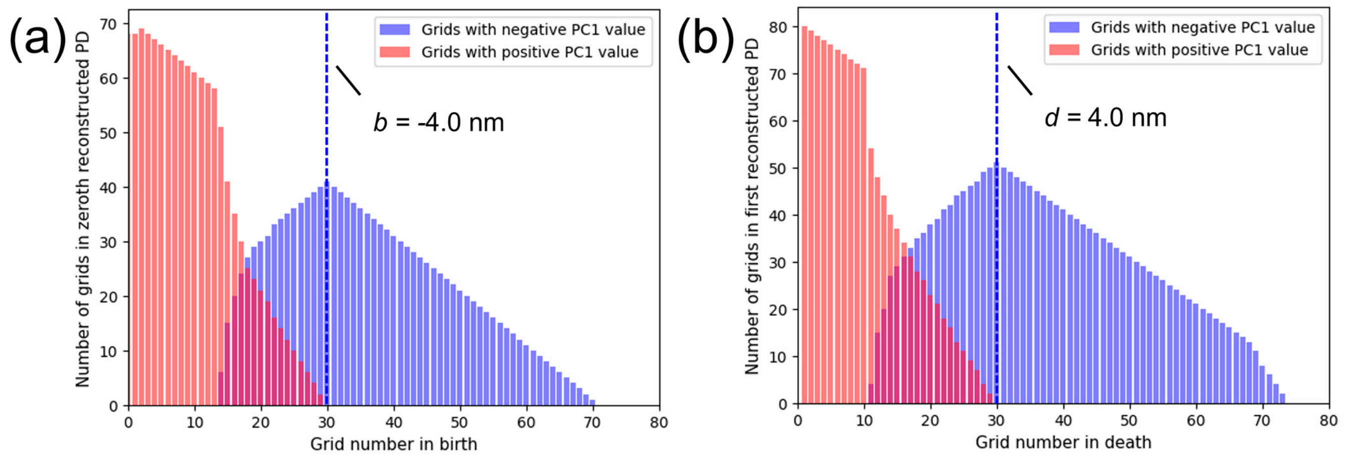

Figure S7. Frequency distributions of the number of grids with negative (blue) and positive (red) PC1 values for (a) b in the reconstructed zeroth PD and (b) d in the first PD. The grid number corresponds to from −10 to 6 nm in (a) and from 10 nm to −6 nm in (b). Only negative PC1 values can be seen at grid number > 30 ($b > -4$ nm) in (a) and grid number > 30 ($d < 4$ nm) in (b). Thus, the characteristic values $b = -4$ nm and $d = 4$ nm can be used to define the blue and non-blue regions for both the zeroth and first reconstructed PDs.

Table S2. Four sample sets for random forest classification and their classification accuracy for each sample set with different synthesis conditions. Each sample set consisted of three nanostructures synthesized under different conditions that were confirmed to be classifiable by hierarchical clustering.

Sample set and synthesis conditions	Accuracy<br>On training set	Accuracy<br>On test set
Sample set1<br>$CO:O_2 = 2:1$<br>500 °C, 600 °C, 700 °C	100%	98.9%
Sample set2<br>$CO:O_2 = 3:1$<br>500 °C, 600 °C, 700 °C	100%	97.9%
Sample set3<br>500 °C<br>$CO:O_2$ = 1:1, 2:1, 3:1	100%	98.9%
Sample set4<br>600 °C<br>$CO:O_2$ = 1:1, 2:1, 3:1	100%	100%

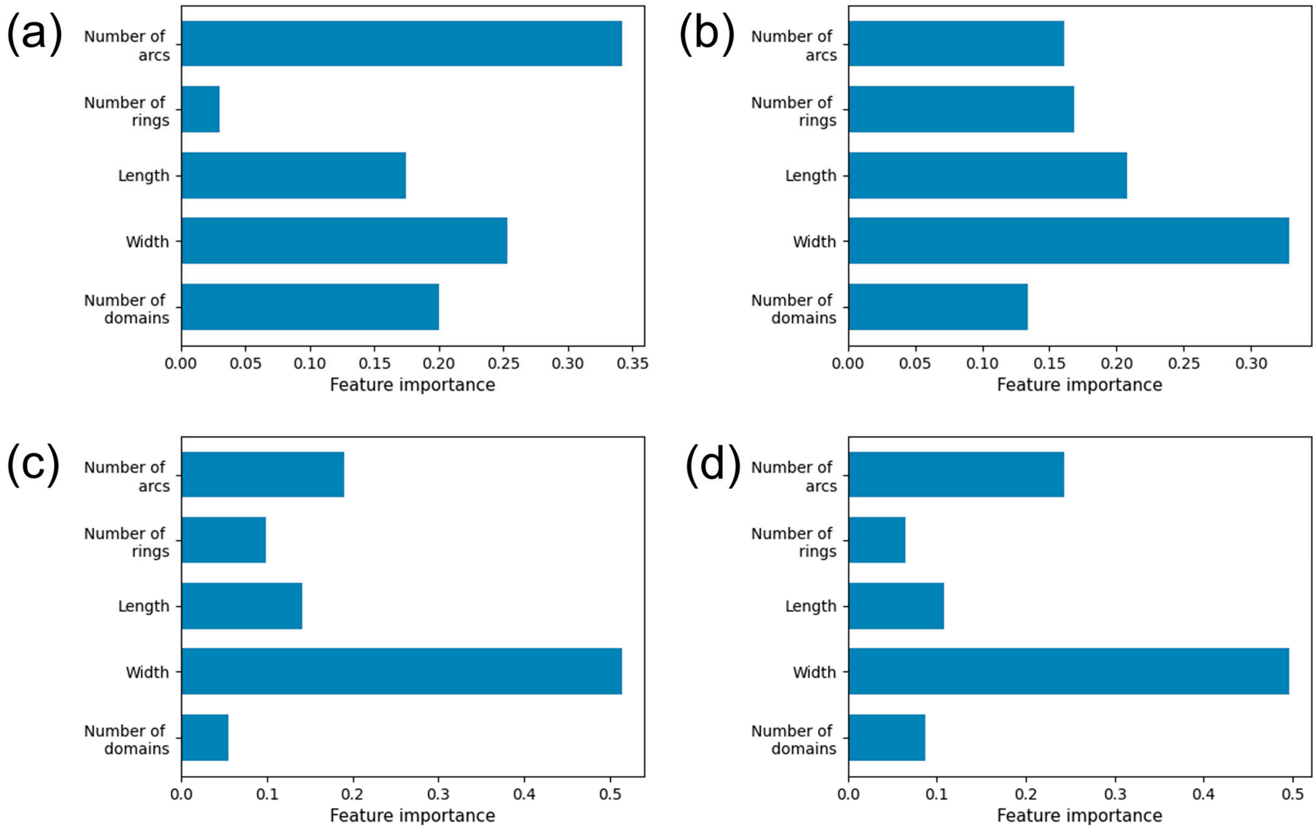

Figure S8. Feature importance for sample set (a) 1, (b) 2, (c) 3, and (d) 4 in Table S2 determined from random forest classification. The number of small arcs and width of CeO_2 phases were the most and second-most important features among the five extracted for the classification.

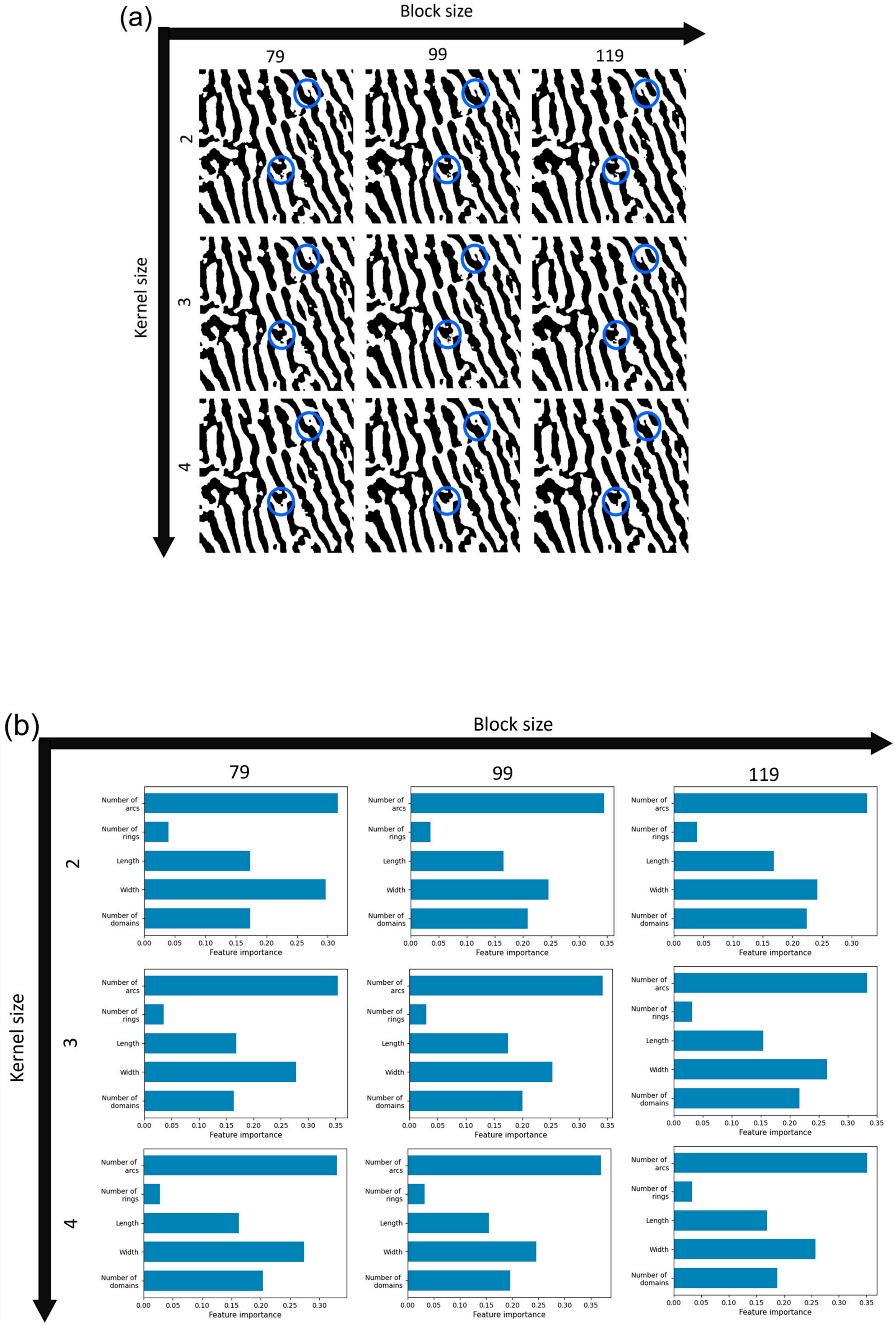
(a)
Block size
79
99
119
Kernel size
2
3
4
(b)
Block size
79
99
119
Kernel size
2
3
4
Number of arcs
Number of rings
Length
Width
Number of domains
Feature importance
0.00
0.05
0.10
0.15
0.20
0.25
0.30
0.35

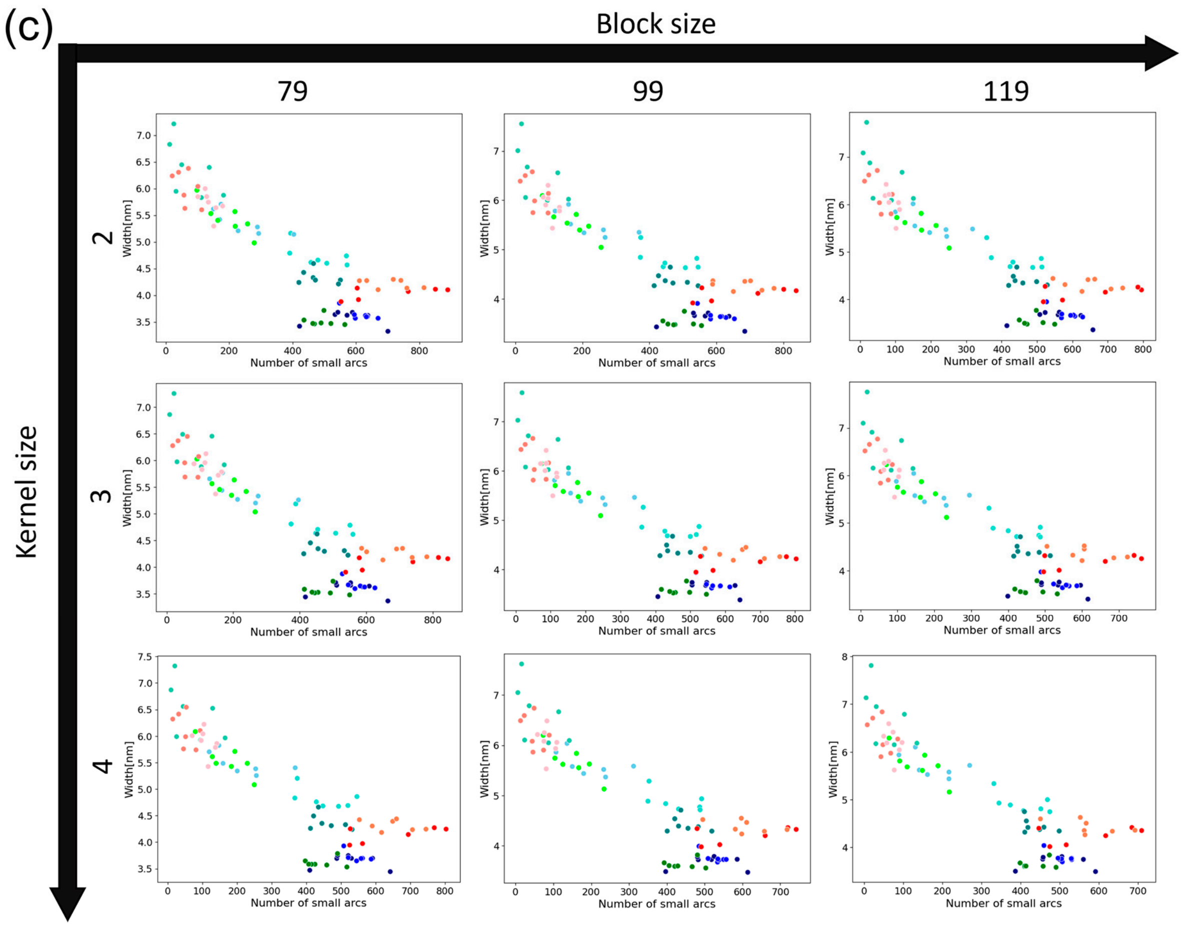

Figure S9. Changes of (a) binarized STEM images of the Pt-CeO_2 nanostructure formed at an annealing temperature of 600 °C and syngas ratio of 2:1, (b) feature importance in random forest classification, and (c) scatter plots of the width and number of CeO_2 phases caused by the two binarization parameters of block size in the adaptive-threshold method and kernel size in opening–closing processing. In (a), slight changes were observed in the shape of the CeO_2 phase (indicated by blue circles). In (b) and (c), the binarization parameters had little effect on the feature importance and scatter plots for sample set 1 (Table S2).

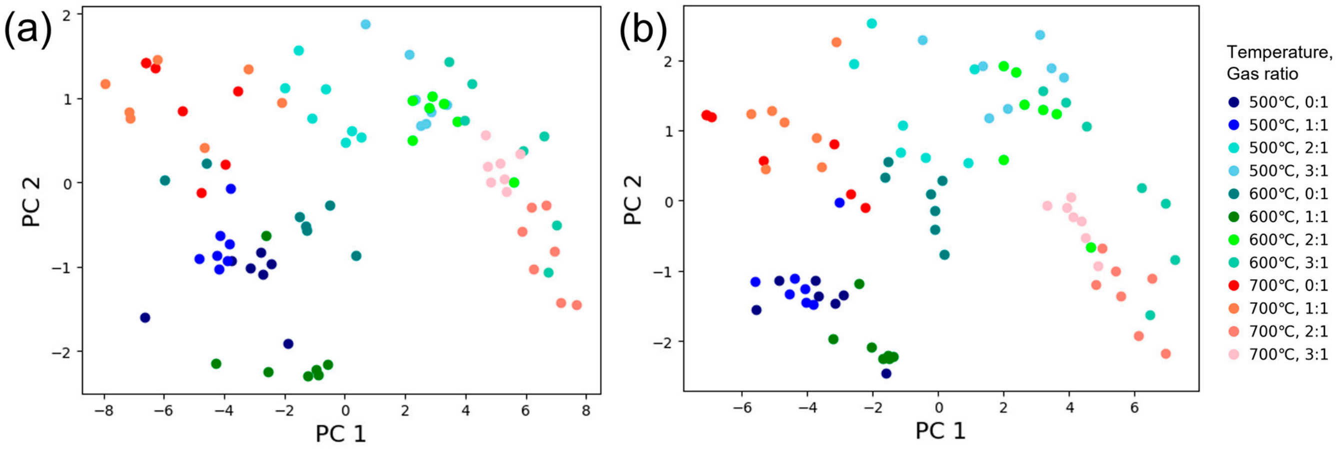

Figure S10. PCA for the (a) zeroth and (b) first PDs of the 12 Pt-CeO_2 nanostructures produced under different synthesis conditions. The first and second principal components obtained from the vectorized PDs have been plotted.
